# Investigation of Halogenated Metallic Phthalocyanine (InPcCl and F_16_CuPc)-Based Electrodes and Palm Substrate for Organic Solid-State Supercapacitor Fabrication

**DOI:** 10.3390/mi16040455

**Published:** 2025-04-11

**Authors:** María Elena Sánchez Vergara, Héctor Iván Sánchez Moore, Luis Alberto Cantera-Cantera

**Affiliations:** 1 Faculty of Engineering, Universidad Anáhuac México, Av. Universidad Anáhuac 46, Col. Lomas Anáhuac, Huixquilucan 52786, Mexico; hector.sanchez@anahuac.mx; 2 Polytechnic University of Cuautitlán Izcalli, Av. Lago de Guadalupe, Colonia Lomas de San Francisco Tepojaco, Cuautitlán Izcalli 54720, Mexico; 3 Departamento de Ingeniería en Control y Automatización, ESIME-Z, Instituto Politécnico Nacional, Av. Luis Enrique Erro S/N, Gustavo A. Madero, Zacatenco, Ciudad de Mexico 07738, Mexico

**Keywords:** organic double-layer supercapacitor, halogen-substituted phthalocyanine, palm substrate, PET substrate, nylon membrane, electrical behavior

## Abstract

In this work, we report on the fabrication of a novel Organic Double-Layer Supercapacitor (ODLSC) using recycled palm as the substrate and electrodes based on halogenated indium and copper phthalocyanines. The electrodes were characterized using Reflectance, the Kulbeka–Munk function, and Fluorescence. Finally, their electrical behavior was evaluated, and the results were compared with those obtained for a more conventional supercapacitor fabricated on polyethylene terephthalate substrate and using indium tin oxide film for electrodes. Based on the experimental measurements of the fabricated ODLSC, the parameter identification of the classical equivalent circuit model was carried out using the Least Squares of Orthogonal Distances (LSOD) algorithm. The results indicated that the palm supercapacitor exhibited behavior more like that of traditional supercapacitors, as the root square mean error (RMSE) values in the model approximation of the experimental data were in the order of $10^{-7}$. Furthermore, the models obtained allowed a determination of the device’s Electrical Impedance Spectroscopy (EIS), revealing that the Palm SC-T1 exhibited capacitive behavior. In contrast, the manufactured Palm SC-T2, PET SC-T1, and PET SC-T2 devices exhibited inductive behavior. All the materials used in this work, such as the substrates, electrodes, separator membranes, and electrolytes, have a high potential to be used in organic supercapacitors.

## 1. Introduction

Supercapacitors (SCs) are becoming increasingly popular in electric vehicles and renewable energy storage applications [1,2,3]. This increase is due to the great promise they claim to have as energy-storage devices with a long life cycle, high power density, fast charge and discharge cycles, as well as low maintenance costs, all in comparison to other conventional energy sources such as batteries or fuel cells [4,5,6,7]. The operation of the energy storage mechanism of SCs is based on the accumulation of charge or reversible redox reactions [8], which also gives excellent potential for use and flexibility, especially in devices that need short energy pulses like wearable electronic devices, electronic paper, or electronic skin [5,9]. Not all SCs are the same; traditional SCs can be categorized into three types depending on their energy storage properties: electrical double-layer capacitors (EDLC), pseudo capacitors, and hybrid supercapacitors. The EDLCs store charges by using an electrical double-layer on the electrode surface, whereas the pseudo capacitors store charges just in a limited area on the surface, and the hybrid supercapacitors are a combination of the mechanism of energy storage of both supercapacitors previously mentioned, providing intermediate performance [8,10,11].

Mathematical models of supercapacitors are widely used for their design, analysis of electrical behavior, state-of-charge and state-of-health estimation, and the development of controllers. The most common approaches in the literature include electrochemical models, equivalent electrical circuit models, and fractional-order models, which accurately describe the system’s electrical variables. Additionally, using neural networks has led to the emergence of intelligent models for predicting the dynamics of energy storage systems based on supercapacitors [1,3,12]. Due to the simplicity of their analysis through linear ordinary differential equations, equivalent electrical circuit models of supercapacitors are widely used to describe their electrical behavior. These models are based on RC networks and are classified into three types: classical, dynamic, and transmission, each with specific applications in the characterization and simulation of supercapacitors [1,12,13]. However, before using any mathematical model, it is essential to address the parameter identification problem, which involves determining the optimal values of the model parameters to minimize the difference between its response and the experimental data obtained from the system for a given input [14,15,16]. This problem is primarily solved using the Least Squares (LS) method and its variations. In the specific case of the parametric identification of equivalent electrical circuit models of supercapacitors, reference [13] demonstrates that the Least Squares of Orthogonal Distances (LSOD) algorithm offers significant advantages over the traditional LS and Least Absolute Deviations (LAD) methods, particularly in identifying the classical, dynamic, and transmission models of supercapacitors.

It is essential to consider the growing need to create more efficient SCs and greener and more sustainable ones due to pollution and energy consumption, which continues to increase. Organic supercapacitors are a promising alternative to traditional SCs due to their potential for low-cost, lightweight, and optimizable synthesis parameters, their redox potential, and their flexible designs [17,18]. The distinction between organic supercapacitors and traditional SCs is that they depart in terms of the organic materials they use in their structure, such as conducting polymers like polyaniline, polypyrrole, or polythiophenes [19,20], and carbon-based materials like graphene, carbon nanotubes/fibers, or activated carbon that are used for the electrodes [19,21]. These materials offer a high surface area and good electrical conductivity, are inexpensive, easy to obtain from various earth-abundant elements, and are easy to synthesize and control [22]. It is important to emphasize that the proper functioning of organic SCs, charge storage, and mechanical resistance under service conditions depends mainly on the materials and substances used as electrodes, electrolytes, and substrates. Thus, the development of high-performance organic materials is essential in this type of solid-state device, and, in addition, a critical approach that is currently being sought is related to the use of recycled or low environmental impact materials, such as Tetra Pak [23], waste newspaper [24], or cotton [25].

Based on the above, the objective of this work is to manufacture new organic SCs using silver paint coated with indium phthalocyanine (InPcCl) and copper phthalocyanine (F_16_CuPc) halogenated as electrodes and tetrabutylammonium hexafluorophosphate (TBAPF_6_) as a supporting electrolyte, supplied through a polymeric membrane of Nylon 11. In addition to these innovative materials, a degradable substrate such as recycled palm is proposed. The decreasing ease of mining fossil fuels, the increasing global energy demand, and increased environmental awareness have led to using and producing materials from sustainable and renewable resources. These palm leaves are composed of cellulose, hemicellulose, and lignin, which also have some interesting mechanical properties, like a tensile strength of 42 MPa, a flexural strength of 63 MPa, and an impact strength of 37.2 kJ/m^2^; these properties represent a significant benefit in the use of the devices [26]. Cellulose in palm leaves is the main structural component providing rigidity and strength. Since most SCs are made from carbon derivatives and cellulose contains a significant amount of carbon, palm leaves are considered a suitable option. Regarding electrode materials, the metallic phthalocyanines used in this study contain halogenated substituents. This is because halogen-substituted phthalocyanines can be prepared as thin films with high charge carrier mobility, making them suitable for applications in electronic devices. Halogen-substituted phthalocyanines are chemically stable and allow for gas-phase transition, making it possible to obtain film structures with physical vapor deposition. This capability significantly enhances production efficiency for low-cost semiconducting and optoelectronic technology [27]. The high electronegativity of halogens such as F or Cl can withdraw electron density from the phthalocyanine ring, stabilizing the central metal’s oxidized states and aiding charge transport. It can increase electrochemical reversibility in energy storage systems requiring multiple redox cycles without degradation. However, to our knowledge, halogen-substituted phthalocyanines have not been widely studied as components of SCs. Hybrid materials with phthalocyanines have been studied mainly, and these can be in the form of nanostructures; some examples are as follows: co-phthalocyanine has been studied as a component of a hybrid SC when it is found in the nanocomposite PcCo@CNTs [5]; ball-type metallophthalocyanines (BTMPc) of Cu, Co, Ni, and Zn have also been studied as electrodes in energy storage systems [6]; and Qi et al. [7] studied tetranitro-substituted cobalt phthalocyanine nanocomposites immobilized on reduced graphene oxide as a supercapacitor electrode hybrid material. Halogenated phthalocyanines such as AlPcCl [27,28], SiPcCl_2_ [29], InPcCl [27,30], MnPcCl [27,31], and TiPcCl_2_ [27] have been studied by some authors of this work as organic semiconductors in optoelectronic devices such as OLEDs, photodiodes, or organic solar cells.

Notably, the chloride mono-axially substituted in the InPcCl stabilizes the phthalocyanine’s structure by reducing its chemical reactivity. Altering the electron density in the indium atom modifies the delocalization of charges throughout the molecule, affecting its optoelectronic properties [32]. On the other hand, fluoride increases phthalocyanine’s thermal and chemical stability due to fluorine atoms in the peripheral rings of the F_16_CuPc molecule. Introducing fluorine substituents leads to a decrease in the electron density of the macrocycle and an increase in the oxidation potential of the MPc molecule [33]. Also, fluoride allows phthalocyanine to absorb light and transport charges better; F_16_CuPc has a relatively high mobility of charge carriers and an n-type semiconducting nature [34]. For this reason, in this work, we propose the incorporation of InPcCl and F_16_CuPc as electrodes in SCs since it is essential to consider that even with all the characteristics and advantages mentioned above, very little research has been conducted on the development of supercapacitors. The main novelty of this work is in demonstrating that efficient solid-state organic SCs can be fabricated using recycled palm leaf as a substrate and metallic phthalocyanines as electrodes. Furthermore, a separating layer made of nylon, which acts as a dispersion membrane for the supporting electrolyte, is also an original proposal for these supercapacitors. To our knowledge, there have been no reports of an Organic Double-Layer Supercapacitor (ODLSC) made with these materials.

## 2. Materials and Methods

**Figure 1:**
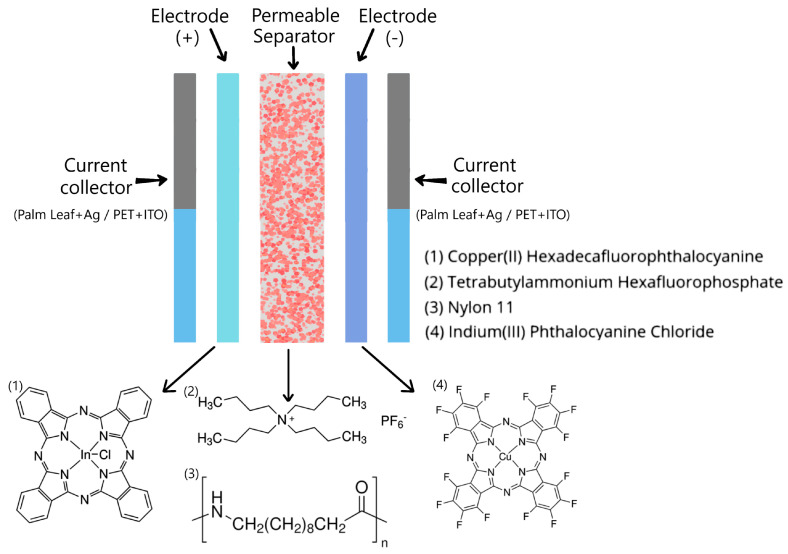
Schematic representation of Organic Double-Layer Supercapacitors.

The InPcCl (chloride indium(III) phthalocyanine: C_32_H_16_ClInN_8_), Nylon 11 (polyundecanolactam: [-NH(CH_2_)10CO-]_n_), F_16_CuPc (copper (II) hexadecafluorophthalocyanine: C_32_CuF_16_N_8_), and TBA·PF₆ (tetrabutylammonium hexafluorophosphate: (CH_3_H_2_CH_2_CH_2_)_4_N(PF_6_)) did not need to be purified before usage and were acquired straight from commercial sources (Sigma-Aldrich, Saint Louis, MO, USA). The two ODLSCs shown in Figure 1 were manufactured, and a commercial palm leaf and PET were used as a substrate. Optimized Ag electrodes with InClPc and F_16_CuPc coatings were used for the SC with the palm substrate. Initially, the palm was cut, sanded, and cleaned to obtain a smooth surface, upon which conductive silver paint was applied. The paint was dried under ambient conditions and vacuumed to ensure complete solvent removal. Using a vacuum sublimation technique, the InClPc and F_16_CuPc coatings were deposited on the Ag. The Nylon 11 separator membrane and the TBA·PF_6_ electrolyte were also deposited using a high-vacuum thermal sublimation system. This technique provided a high degree of variable control during deposition, including time, sublimation temperature, substrate temperature, and base pressure. The optimization of these variables allowed for the film’s deposition with a precise thickness and molecular orientation. For the InClPc and the Nylon 11, an 8.6 × 10^−5^ Torr vacuum was used; for the TBA·PF_6_ and the F_16_CuPc, an 8.6 × 10^−6^ Torr vacuum was used. Thicknesses were measured with a quartz crystal microbalance monitor connected to a thickness sensor. The measured thicknesses were InPcCl = 904 Å, Nylon 11 = 186 Å, TBA·PF₆ = 116 Å, and F_16_CuPc = 470 Å.

The ODLSC on PET had important differences concerning the SC manufactured on palm (see Figure 1). For this device, the significant difference was, on the one hand, the change in substrate to commercial polyethylene terephthalate (PET), and one of the electrodes was composed of indium tin oxide film (ITO: In_2_O_3_/(SnO_2_)_x_) coated with an InClPc film. The second electrode had the same conformation as in the first device, ITO coated with F_16_CuPc, and the Nylon 11 was included as a structural intermediate layer for the inclusion of the TBA·PF_6_. The thicknesses of these materials were the same as those of the palm-mounted device. This was because both SCs were manufactured simultaneously. The devices were treated by thermal relaxation in a Briteg SC-92898 (Instrumentos Científicos, S.A de C.V., México City, México) oven at 140 °C for 12 min. Thermal relaxation was performed to introduce the supporting electrolyte into the nylon membrane and promote charge transport in the supercapacitor. An IR spectroscopy analysis was performed using a spectrometer Nicolet iS5-FT (Thermo Fisher Scientific Inc., Waltham, MA, USA) to verify the main functional groups in phthalocyanines and check whether decomposition occurred during their deposition. IR spectroscopy was carried out on the phthalocyanines used as electrodes, which were previously deposited on silicon, simultaneously using the same vacuum sublimation system and the same operating parameters as were used when deposited on the palm and PET of the ODLSCs. The Reflectance and Kubelka–Munk function were obtained using a Thermo Scientific Evolution 220 UV-Vis spectrophotometer in a wavelength range from 190 to 1100 nm (Thermo Fisher Scientific Inc., Waltham, MA, USA). Fluorescence was evaluated on an FP-8550 spectrofluorometer (Jasco International, Tokyo, Japan). The electrical properties in the ODLSCs were carried out using a sensor station with a Next Robotix lighting controller circuit (Comercializadora K Mox, S.A. de C.V., Mexico City, Mexico) and a Keithley 4200-SCS-PK1 auto-ranging picoammeter (Tektronix Inc., Beaverton, OR, USA).

## 3. Results and Discussion

### 3.1. Characterization of the Electrodes

**Figure 2:**
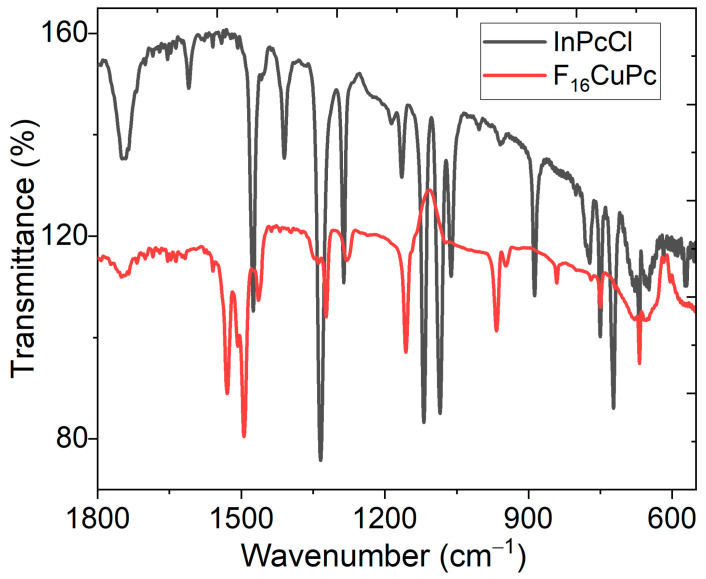
IR spectra of InPcCl and F_16_CuPc electrodes in film.

The IR analysis of the electrodes helps to find functional groups on the electrode’s surface and determine whether chemical decomposition of the phthalocyanines occurs during their deposition [23]. Furthermore, the InPcCl and F_16_CuPc films possess unique properties, including strong absorption, emission, and photostability. This is because the molecular orientation and stacking of the phthalocyanine molecules are related to their optical behavior due to the different monoclinic (α-phase) and triclinic (β-phase) forms that they can acquire, which can be monitored by IR spectroscopy. A band can identify the α-phase and the β-phase bands around 720 cm^−1^ and around 778 cm^−1,^ respectively [35,36]. Figure 2 presents the IR spectra for the InPcCl and F_16_CuPc films, and Table 1 presents the most representative signals of phthalocyanines in film, which are compared to their signals in KBr pellets. The mode at 1608 cm^−1^ in the spectrum of InPcCl attributed to C=C stretching shifts to 1618 cm^−1^ for F_16_CuPc, the peaks at 1749 ± 1 cm^−1^ are identified as a C-H stretch, and the C-C benzene stretch is associated with the peaks observed at 1542 and 1475 cm^−1^ for the InPcCl and at 1530 and 1495 cm^−1^ for F_16_CuPc. The signals at 1328 ± 6 and 1414 ± 5 cm^−1^ verify in-plane pyrrole and isoindole stretch, respectively. According to IR spectroscopy, two relevant results are obtained: on the one hand, phthalocyanines are not chemically degraded when deposited as electrodes. This is confirmed by the similarity of the signals in Table 1 for pellets and the film; the small shifts between the values are due to the residual stresses generated in the phthalocyanine molecules during their deposition as films on the substrate. On the other hand, in the electrodes, InPcCl is present in a combination of the α-phase and the β-phase, and F_16_CuPc is present only in the β-phase.

**Figure 3:**
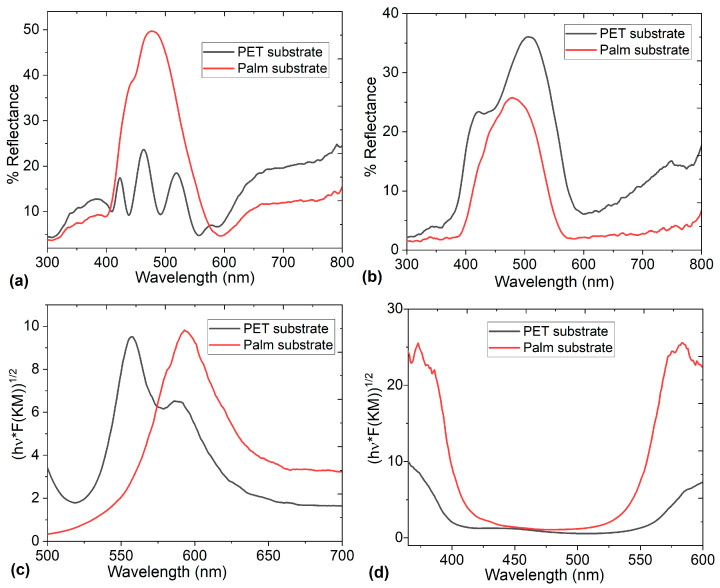
The Reflectance of (**a**) InPcCl and (**b**) F_16_CuPc electrodes on PET and palm substrates. The Kulbeka–Munk function of (**c**) InPcCl and (**d**) F_16_CuPc electrodes on PET and palm substrates.

InPcCl and F_16_CuPc are phthalocyanines that absorb visible light radiation and exhibit high optical stability. For this reason, the electrodes are studied in terms of their Reflectance R, since if the ODLSC is used in optical or photovoltaic devices, a low R is required to help avoid energy loss through reflected light, maximizing the device efficiency. Figure 3a,b show the spectra for the electrodes on PET and palm substrates, and it is evident that R changes significantly when the electrode type and substrate are modified. The electrode with InPcCl has the lowest R (Figure 3a) when it is deposited on PET-ITO substrate, while the electrode with F_16_CuPc has the lowest R (Figure 3b) when it is deposited on palm-Ag substrate. However, the maximum R obtained for each electrode and recorded in Table 2 is low for the two electrodes with F_16_CuPc and low for the electrode with InPcCl. It is evident that even the F_16_CuPc electrode on the palm, with 50% maximum Reflectance, has low Reflectance, and the rest of the electrodes, with Reflectance between 24 and 36%, could be used in SCs connected to optical or photovoltaic devices. Additionally, it is crucial to consider that in all spectra, the Q-band is distinguished at wavelength λ, between 400 and 600 nm, corresponding to the visible region of the spectrum and being associated with the electronic transitions π→π* of the 18 conjugated π electrons of the structure of the phthalocyanines InPcCl and F_16_CuPc. The Q-band refers to the electronic transitions of the highest occupied molecular orbital (HOMO) to the lowest unoccupied molecular orbital (LUMO) of the Pc ring and is very useful, since these transitions increase the electronic conductivity, which is key to improving the performance of F_16_CuPc and InPcCl electrodes. The presence of the Q-band facilitates the participation of π electrons in reversible redox processes, which could occur in the electrodes and increase their specific capacity. This results in a higher energy density in pseudocapacitive systems than in purely electrostatic supercapacitors. Furthermore, the electron delocalization associated with the Q-band could allow phthalocyanines to form stable complexes with the TBA·PF₆ electrolyte. This favors charge storage mechanisms, especially in pseudocapacitive supercapacitors, where surface redox reactions occur. Although the Q-band of phthalocyanines is known for its role in optical applications, in ODLSCs, it could contribute to the improved electron transport, charge storage capacity, and redox stability of the F_16_CuPc and InPcCl electrodes [37]. It is important that even on palm, phthalocyanines do not lose their charge transport capacity and their optical behavior, thanks to their Q-band, which maintains its shape and intensity when compared to pristine halogenated phthalocyanine films [27,28,29,30,31]. According to Qi et al. [7] and Wang et al. [38], the presence of phthalocyanines and optical behavior in supercapacitor electrodes can be demonstrated by the characteristic absorption of the Q-band. Another important aspect is the red shift that appears in the spectra when comparing the curves for each substrate. In the case of InPcCl (Figure 3a), the wavelength at maximum Reflectance is 506 nm for PET and shifts to 479 nm for palm. In the case of F_16_CuPc (Figure 3b), Reflectance is from 518 nm in PET and shifts to 476 nm in palm. These shifts suggest interfacial interactions between the phthalocyanine, ITO, and Ag of PET and palm, respectively [38].

As mentioned above, the Q-band provides information on the transport of charges from the HOMO to the LUMO in phthalocyanines, which refers to the optical band gap. This parameter is considered necessary, because if the electrodes have a low band gap, a higher conductivity will be generated, which is related to a lower internal resistance of the electrodes and an improvement in the charge transfer during the charge/discharge cycles. The band gap for the electrodes is calculated using Reflectance measurements by the Kubelka–Munk function F(K-M) [39] and from the Tauc model with Equation (1) [39,40,41]:

(1) $$h\nu \alpha =A{\left(h\nu -E_{g}\right)}^{p},$$

where hν is the photon energy (h = Planck’s constant y ν = 1/λ); A is a proportionality constant; E_g_ is the energy band gap; and the exponent p depends on the band structure of the semiconductor, and this value is 2 for indirect transitions, where the participation of phonons is required [39]. The absorption coefficient α is directly proportional to F(K-M), and in the Tauc equation, α can be replaced by this function as follows in Equation (2):

(2) $$\left(h\nu \times F(KM)\right)=A{\left(h\nu -E_{g}\right)}^{p},$$

The procedure consists of plotting the (hν × F(KM))^1/2^ versus λ fitting (Figure 3c,d) the linear portion of this curve with a straight line; the intercept and the use of Equation (3) provide the band gap value (c = light speed). Table 2 presents the band gap values for each electrode on the palm and PET.

(3) $$E_{g}=\frac{hc}{\lambda },$$

According to the values shown in Table 2, the electrode with InPcCl presents a much lower band gap than the electrode with F_16_CuPc. This is due to the structure of each phthalocyanine. The indium and its coordination with the chlorine atom introduce electron-donating characteristics, and the electron density is more concentrated around the macrocyclic ring. This raises the energy of the HOMO, narrowing the band gap by bringing it closer to the LUMO. Additionally, the indium atom has a larger ionic radius and induces more π electron delocalization in the phthalocyanine ring, leading to a smaller band gap. On the other hand, F_16_CuPc has 16 fluorine atoms attached to the phthalocyanine ring; fluorine is an electron-withdrawing group, which lowers the HOMO energy level and stabilizes the LUMO, and this widens the band gap. On the other hand, copper ions stabilize the π system under the influence of the fluorine substituents, and the fluorine atoms pull electron density away from the core, resulting in a higher band gap. The differences in the optical band gaps of the two phthalocyanines and their behavior as electron donors of InPcCl and electron acceptors of F_16_CuPc are an indication of their adequate capacity as a pair of electrodes to generate the charge transport that must be carried out in the supercapacitor, regardless of whether the substrate is palm-Ag or PET-ITO. On the other hand, it is important to consider that the band gap values obtained for the electrodes are within the range of organic semiconductors [42], which enhances their use not only in SCs but also in optoelectronic devices such as light-emitting diodes, electrochromic devices, and solar cells [43].

**Figure 4:**
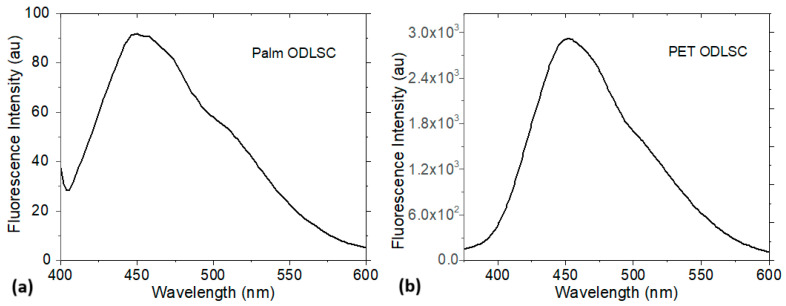
Fluorescence intensity of ODLSs on (**a**) palm substrate and (**b**) PET substrate.

The Fluorescence spectra in Figure 4 are obtained for the two supercapacitors at 382 nm excitation. All maximum emissions of wavelengths around 450 nm correspond to blue light wavelengths, and the PET supercapacitor exhibits the highest emission intensity. This high Fluorescence is advantageous for their use with devices such as organic light-emitting diodes (OLEDs). These devices can efficiently emit light when an electric current is applied to them, or when light excites them. Another advantage is that the high Fluorescence can indicate the quality of the phthalocyanine in the electrode, which can facilitate the detection of changes in the electrode state during charging and discharging, improving the monitoring and control of the supercapacitor. The Stokes shift (Δ_Stokes_), which is the difference in energy or wavelength between the absorbed light and the light emitted by the phthalocyanines at the electrodes after being excited, is 18.5 eV (67 nm) for the supercapacitor on palm and 17.7 eV (70 nm) for the supercapacitor on PET.

### 3.2. Parameter Identification of Organic Supercapacitor Devices

**Figure 5:**
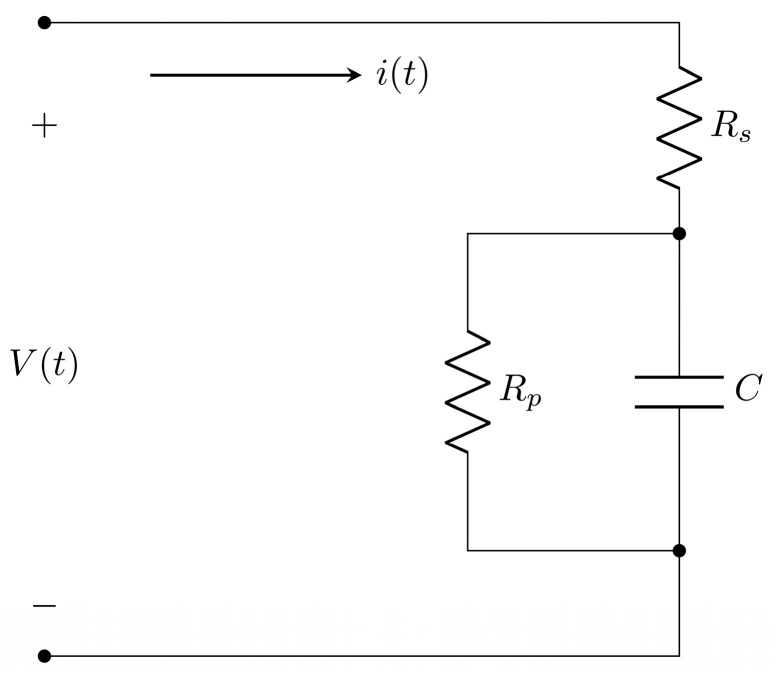
Supercapacitor classic model.

The diverse literature, such as [44,45,46], presents studies and methods for fabricating supercapacitors using organic materials. However, parameter identification is a fundamental technique widely used for characterizing equivalent RC circuit models in inorganic supercapacitors [13]. It enables the analysis of their behavior under different input signals and design control strategies for charging and discharging processes. This section identifies the classical RC model to represent the electrical dynamic of the organic supercapacitor manufactured. The supercapacitor electrical dynamics can be modeled through linear equivalent electric circuit models, such as the classic, dynamic, and transmission circuits, which are compounded by RC networks [13]. These models are represented in linear ordinary differential equations, in which the model parameters must also be identified to ensure that the models accurately describe experimental data. Depending on the equation order, the parameter identification problem becomes more difficult, because more derivatives of the experimental data are necessary. Since the number of RC networks defines the equation order, the classic circuit is the simplest model to describe the electrical dynamics of supercapacitors because it has only one RC network. The classic model of the supercapacitor equivalent electrical circuit is shown in Figure 5.

**Figure 6:**
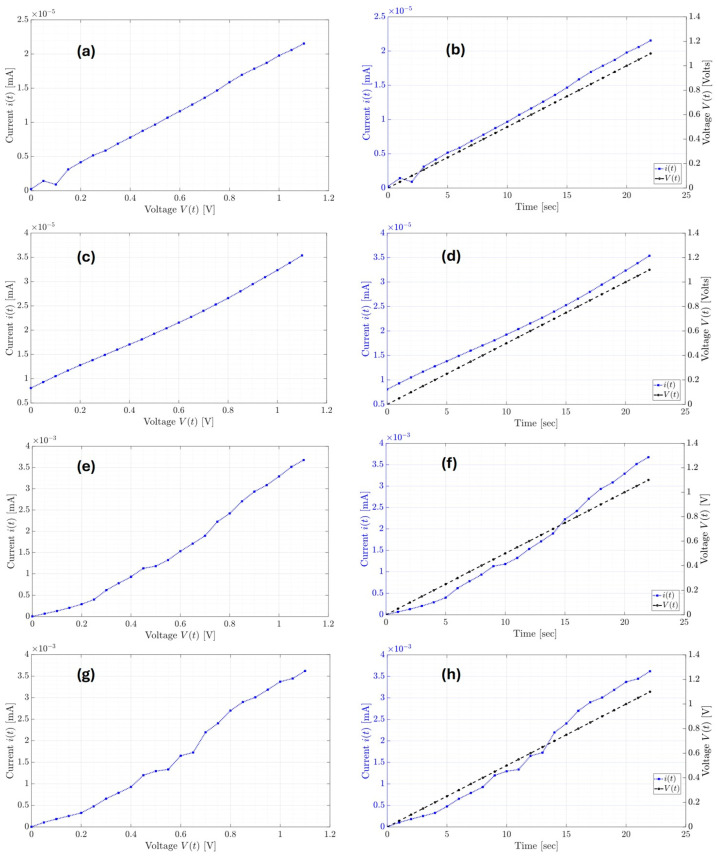
V-I curves of the (**a**) Palm SC-T1, (**c**) Palm SC-T2, (**e**) PET SC-T1, and (**g**) PET SC-T2, and the organic supercapacitor devices’ input voltage and output current time series in (**b**) Palm SC-T1, (**d**) Palm SC-T2, (**f**) PET SC-T1, and (**h**) PET SC-T2.

**Figure 7:**
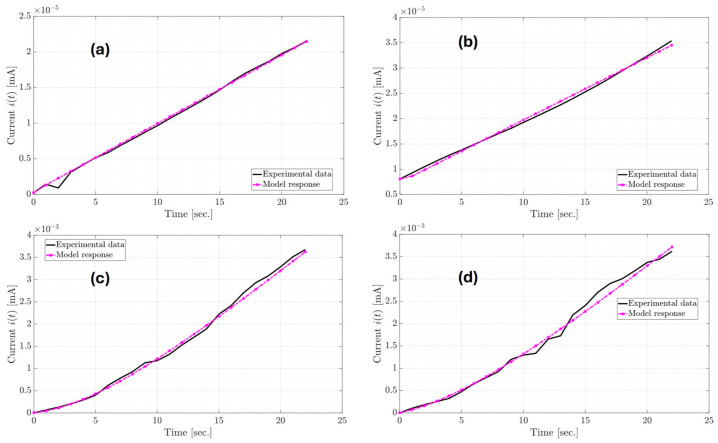
Current responses of the organic supercapacitor equivalent circuit classic models in (**a**) Palm SC-T1, (**b**) Palm SC-T2, (**c**) PET SC-T1, and (**d**) PET SC-T2.

The transfer function of the classic model that relates the supercapacitor output voltage $V\left(s\right)$ and supercapacitor input current $I\left(s\right)$ from Figure 5 is given by Equation (4).

(4) $$\frac{V\left(s\right)}{I\left(s\right)}=\frac{b_{1}s+b_{0}}{s+a_{0}},$$

with $b_{1}=R_{s}$, $b_{0}=\frac{R_{s}}{R_{p}C}+\frac{1}{C}$, and $a_{0}=\frac{1}{R_{p}C}$, which are unknown in the models of the manufactured supercapacitors. On the other hand, experimental data are essential to carry out the parameter identification of any mathematical model; in this sense, the experimental data of the manufactured supercapacitors are shown in Figure 6. As shown in Figure 6, the manufactured ODLSCs on palm (Palm SC) and PET (PET SC) are supplied with a voltage from 0 to 1.1 V. Two experiments are conducted on each supercapacitor: one immediately after applying the electrolyte and another 10 min after its application. Figure 6a,c show the V-I curves for the first and second experiments of the Palm SC device, denoted by Palm SC-T1 and Palm SC-T2, respectively. On the other hand, Figure 6b,d show the voltage and current time series for the Palm SC-T1 and Palm SC-T2 experiments, respectively. Regarding the PET SC-T1 and PET SC-T2 experiments, Figure 6e,g present the V-I curves, and Figure 6f,h show the voltage and current time series, respectively. Using the voltage and current time series of the SC devices in Figure 6 and the Least Squares of Orthogonal Distances (LSOD) method [13,47], the estimates of the parameters $b_{1}$, $b_{0}$, and $a_{0}$ from the supercapacitor classic model in Equation (4) and the approximation’s root square mean error (RMSE) for all experiments are shown in Table 3. The current responses of the manufactured organic supercapacitor equivalent circuit classic models to the input voltages from 0 to 1.1 V using the parameters from Table 3 are shown in Figure 7.

The model responses from Figure 7a,b correspond to the current dynamics of the Palm SC-T1 and Palm SC-T2 devices, respectively. In contrast, the model responses from Figure 7c,d correspond to the current responses of the PET SC-T1 and PET SC-T1 devices. From the RMSE in Table 3, better approximations of the models are carried out with the Palm SC device compared to the approximations of the PET SC device. Still, in general, all approximations are acceptable. On the other hand, it is important to mention that the estimates in Table 3 differ for each manufactured supercapacitor device and test. This means that the performance of the supercapacitor has changed due to the diffusion of the electrolyte inside the device. The estimates presented in Table 3 show the values of the lumped parameters. However, when broken down in terms of the components of the classic equivalent circuit of a supercapacitor, the values detailed in Table 4 are obtained.

**Figure 8:**
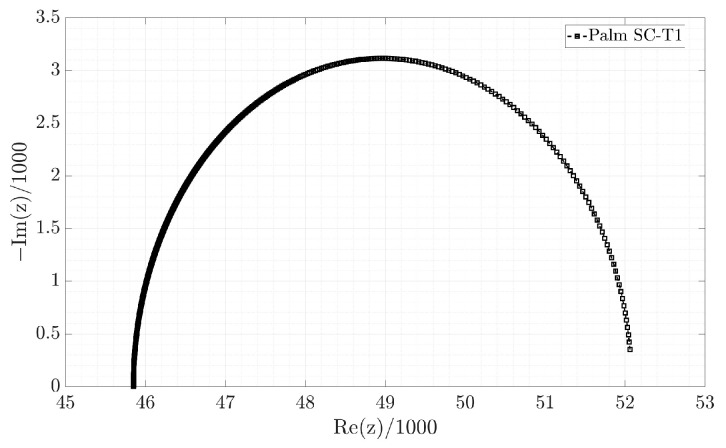
EIS of the classic equivalent circuit model of the Palm SC-T1 device.

**Figure 9:**
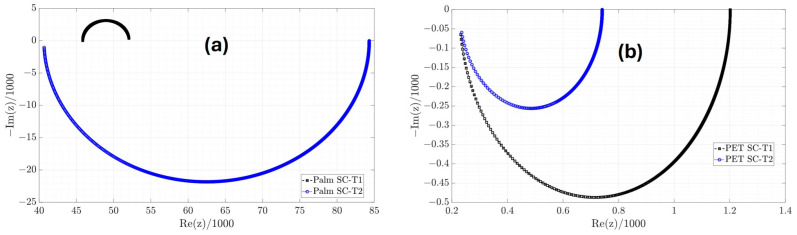
EIS of the classic equivalent circuit model of the (**a**) Palm and (**b**) PET devices.

From the estimates in Table 3, the parameters of the classic equivalent circuit in Table 4 are obtained; however, only the Palm SC-T1 device receives consistent values for the C capacitor and the $R_{s}$ and $R_{p}$ resistances because they are positive. In the other devices, the values of C and $R_{p}$ are negative. Using the classic equivalent circuit model of the Palm SC-T1 device, the Electrical Impedance Spectroscopy (EIS) of the manufactured supercapacitor from 0.05 to 10,000 rad/s with steps of 0.01 is shown in Figure 8. EIS is a technique used to study the electrical properties of supercapacitors. This technique applies a low-amplitude alternating (AC) voltage signal and measures the current response. One of the standard graphs of EIS analysis is the Nyquist diagram (Figure 8), which represents the imaginary versus real parts of the impedance and helps to interpret resistances, capacitances, and charge transfer processes. Additionally, the 6 mΩ diameter of the semicircle represents the charge transfer resistance (Rct). On the other hand, the EIS graphs for the Palm SC-T2, PET SC-T1, and PET SC-T2 are shown in Figure 9. Figure 8 and Figure 9a show that the only device exhibiting capacitive behavior is the Palm SC-T1, whose EIS graph is in the first quadrant. In contrast, the EIS graphs of the Palm SC-T2, PET SC-T1, and PET SC-T2 devices in Figure 9a,b are in the fourth quadrant, indicating inductive behavior. Therefore, the Palm SC-T1 manufactured device exhibits behavior more consistent with a supercapacitor than the other manufactured devices.

## 4. Conclusions

Two Organic Double-Layer Supercapacitors (ODLSCs) were manufactured using InPcCl and F_16_CuPc electrodes deposited on Ag- and ITO-coated palm and PET substrates, respectively. The electrodes were structurally characterized using IR spectroscopy. The results demonstrated that the high-vacuum sublimation method used to deposit the phthalocyanine electrodes did not alter their chemical composition. The Reflectance of the electrodes was also measured and was found to be less than 50%, which is a good result for SCs used with organic optoelectronic or photovoltaic devices. Additionally, the band gap was calculated using the Kubelka–Munk function. The InPcCl electrode exhibited a band gap ranging from 1.86 to 1.93 eV, while the F_16_CuPc electrode had a band gap of 3.05 eV. This difference arose from each phthalocyanine’s structure and the electron donor or acceptor properties, facilitating charge transport within the supercapacitor. The optical gap values obtained placed InPcCl and F_16_CuPc as organic semiconductors. The electrodes exhibited Fluorescence at 382 nm excitation, and their maximum emission was found at wavelengths around 450 nm, corresponding to the blue region of the electromagnetic spectrum. Based on the electrical behavior of the fabricated Organic Double-Layer Supercapacitor, the parametric identification of the classical equivalent circuit model was performed for each device. Regarding the approximation of the models to the experimental data, the RMSE values ranged from $10^{-4}$ to $10^{-7}$, indicating a good agreement between the models and the current data. However, in each test performed, the model parameters varied, suggesting that electrolyte diffusion within the supercapacitor affected the current conduction dynamics. On the other hand, when solving the system of equations to determine the equivalent circuit parameters, only the Palm SC-T1 device exhibited consistent values for C, $R_{s}$, and $R_{p}$. This allowed for the determination of its impedance spectroscopy (EIS), which indicated that this device exhibited behavior more consistent with that of inorganic supercapacitors. In contrast, the other manufactured devices exhibited indictive behavior. The Nyquist diagram for the supercapacitor manufactured on palm suggested that charge transfer processes occurred, and resistive–capacitive behavior was presented. All the new materials studied in this work, such as substrates, electrodes, separator membranes, and electrolytes, have a high potential to be used in organic supercapacitors. Therefore, device optimization and experimentation under different charge–discharge cycles are left for future work.

## Figures and Tables

**Table 1:** Assignments of the main IR bands for InPcCl and F_16_CuPc films and KBr pellets.

Sample	C=C Benzene Stretch (cm^−1^)	C-C Benzene Stretch (cm^−1^)	Isoindole Stretch (cm^−1^)	In-Plane Pyrrole Stretch (cm^−1^)	C-H Stretch (cm^−1^)	α-Phase (cm^−1^)	β-Phase (cm^−1^)
InPcCl film	1608	1542, 1475	1410	1334	1748	723	774
InPcCl KBr pellet	1608	1541, 1473	1419	1334	1750	726	771
F_16_CuPc film	1618	1530, 1495	1419	1323	1750	-	771
F_16_CuPc KBr pellet	1617	1524, 1505	1417	1319	1748	-	766

**Table 2:** Reflectance, Kubelka–Munk gap, and the Stokes shift in the electrodes.

Electrode	Reflectance Maximum (%)	KM Band Gap (eV)
InPcCl on palm	26	1.86
InPcCl on PET	36	1.93
F_16_CuPc on palm	50	3.04
F_16_CuPc on PET	24	3.04

**Table 3:** Parameters of the classic model’s transfer function.

Parameter	Organic Double-Layer Supercapacitor			
	Palm SC-T1	Palm SC-T2	PET SC-T1	PET SC-T2
$b_{1}$	45,849.53691	84,316.1838	1201.52793	740.939817
$b_{0}$	45,834.70244	84,290.7887	170.674715	97.3210655
$a_{0}$	0.88007207	2.07389841	0.74966497	0.42602235
RMSE	$3.41\times 10^{-7}$	$4.91\times 10^{-7}$	$7.02\times 10^{-5}$	$1.03\times 10^{-4}$

**Table 4:** Parameters of the classic equivalent circuit.

Parameter	Organic Double-Layer Supercapacitor			
	Palm SC-T1	Palm SC-T2	PET SC-T1	PET SC-T2
C [μF]	182.36	−11.04	−1369.73	−4580
$R_{s}$ [kΩ]	45.85	84.32	1.202	0.741
$R_{p}$ [kΩ]	6.23	−43.67	−0.974	−0.512

## Data Availability

Data are contained within the article.
